# Ultrasound-guided axillary vein versus internal jugular vein access for totally implantable venous access ports in breast cancer: a retrospective comparison of patient-reported outcomes

**DOI:** 10.3389/fonc.2025.1684119

**Published:** 2025-11-06

**Authors:** Jianfeng He, Rong Zhang, Tianhong Cai, Kai Chen, Tenghui Zhan

**Affiliations:** 1 Department of Vascular Surgery & Interventional Therapy, Fujian Maternity and Child Health Hospital, Fuzhou, Fujian, China; 2 College of Clinical Medicine for Obstetrics & Gynecology and Pediatrics, Fujian Medical University, Fuzhou, Fujian, China

**Keywords:** axillary vein access, totally implantable venous access port, breast cancer, patient-reported outcomes, comfort, cosmetic result, satisfaction

## Abstract

**Background:**

Patient-centered venous access is critical in breast cancer supportive care. While the tunnel-less axillary vein (AxV) approach for totally implantable venous access port (TIVAP) implantation may improve patient experience, comparative evidence on patient-reported outcomes (PROs) against the standard internal jugular vein (IJV) approach remains limited.

**Methods:**

This single-center retrospective cohort study compared ultrasound-guided IJV (n = 106) versus AxV (n = 102) TIVAP implantation in breast cancer patients (September 2020–February 2025). Primary outcomes included postoperative comfort (assessed at 1 day) and cosmetic outcome and satisfaction (assessed at 6 months). Complications were monitored for 6 months. Group comparisons utilized chi-square/Fisher’s exact tests. To control for potential confounders, multivariable logistic regression analyses were performed, adjusting for age, body mass index, and implantation side. Complications were monitored for 6 months.

**Results:**

The AxV approach significantly enhanced early postoperative comfort, with a higher rate of no discomfort (Grade 0: 72.5% vs. 59.4%, p = 0.032). At 6 months, the AxV approach demonstrated superior, favorable cosmetic outcomes (Grades 1–2: 93.1% vs. 67.9%, p < 0.001) and higher overall satisfaction (94.1% vs. 85.8%, p = 0.039). Multivariable analysis confirmed the AxV approach as an independent predictor for ideal comfort [adjusted odds ratio (aOR) = 4.48, p = 0.0002], favorable cosmetic outcome (aOR = 6.22, p < 0.001), and overall satisfaction (aOR = 3.07, p = 0.033). More AxV patients would choose the port again (83.3% vs. 72.6%, p = 0.045). The overall complication rates were comparable between groups [4.8%, 0.269/1,000 central line-days (CD) vs. 4.9%, 0.279/1,000 CD; p = 0.957].

**Conclusion:**

For breast cancer patients, the ultrasound-guided AxV approach for TIVAP provides superior early postoperative comfort, long-term cosmetic results, and patient satisfaction without increasing early complication risks, representing a significant patient-centered advancement in venous access.

## 1 Introduction

Totally implantable venous access ports (TIVAPs) are crucial for facilitating long-term intravenous therapies, such as chemotherapy and parenteral nutrition, in patients with breast cancer. Conventionally, the internal jugular vein (IJV) and subclavian vein (SCV) have been the preferred access routes. The IJV approach requires three key steps: ultrasound-guided venipuncture, creation of a subclavicular port pocket, and subcutaneous catheter tunneling between these sites. This multi-step process increases invasiveness, frequently causing post-procedural discomfort including odynophagia and neck-motion-related pain, tunnel-site inflammation (1), and an unsightly catheter course beneath the skin (2). The SCV approach, while less traumatic, exposes patients to potentially life-threatening complications—hemothorax, pneumothorax, and pinch-off syndrome (which may culminate in catheter fracture or rupture) (3–5). These risks, especially pinch-off syndrome, which can lead to catheter fracture, have prompted the search for safer alternatives.

With the widespread adoption of ultrasound guidance, a tunnel-less TIVAP technique via the axillary vein (AxV) has emerged as an attractive alternative. This technique, requiring only a single infraclavicular incision, has been associated with improved cosmetic outcomes (6) and enhanced patient comfort, factors of particular importance to female patients.

Traditional comparative studies have predominantly focused on technical success rates and objective morbidity profiles (infection, thrombosis, and catheter malfunction) (7–10). There are many problems of psychological burden in patients with a tumor implanted in the port of intravenous infusion (11). In the era of patient-centered care, however, peri-procedural comfort, scar aesthetics, and overall satisfaction have become essential benchmarks for evaluating therapeutic interventions. This single-center retrospective cohort study aimed to comprehensively compare patient-reported outcomes (PROs), including postoperative comfort, long-term cosmetic results, and overall satisfaction, between the ultrasound-guided IJV and AxV approaches for TIVAP implantation in women with breast cancer.

## 2 Methods

### 2.1 Study population

**Figure 1:**
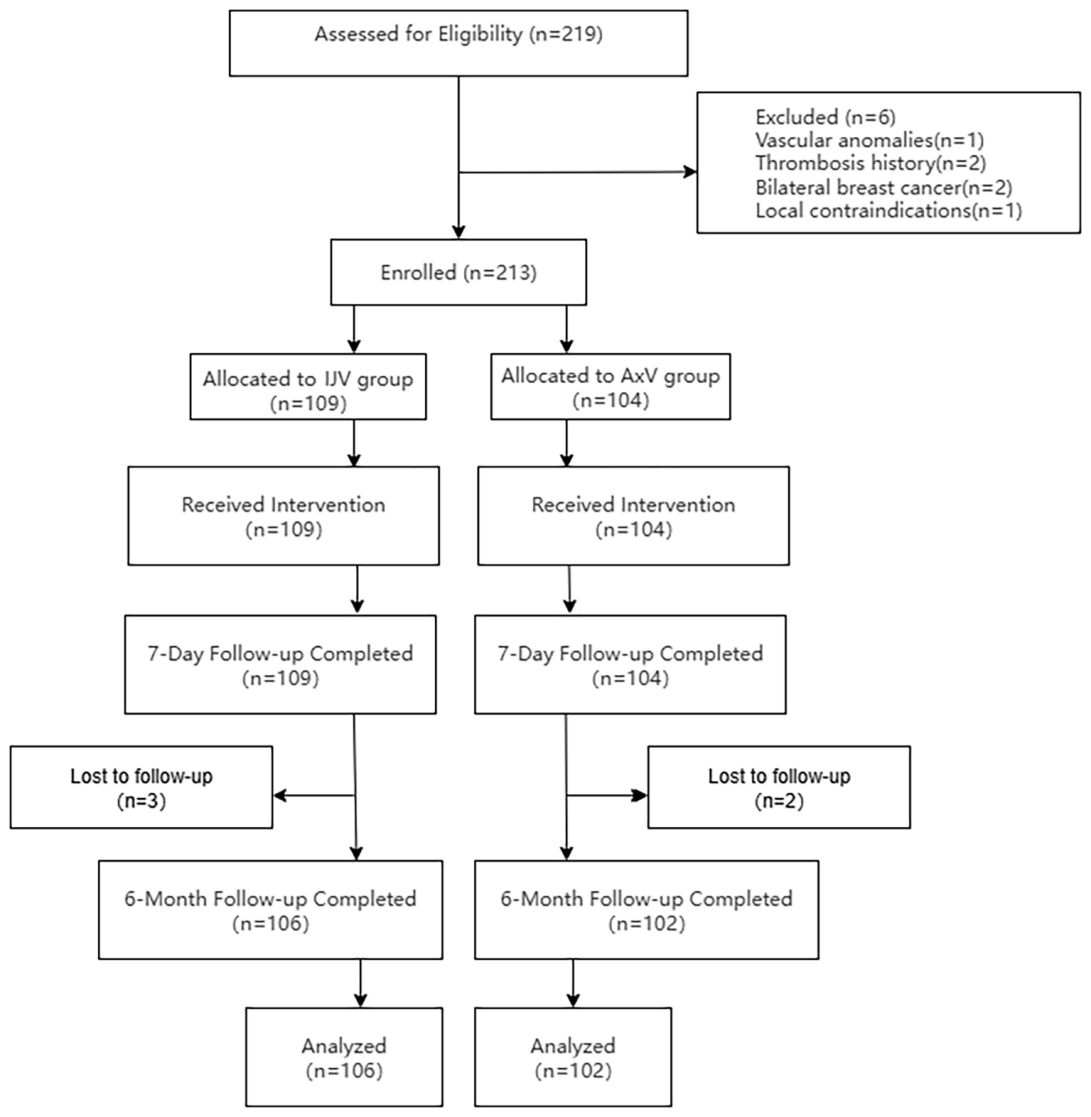
Patient enrollment flowchart.

This retrospective study was conducted in strict adherence to the principles outlined in the Declaration of Helsinki and received ethical approval from the Fujian Maternal and Child Health Hospital Ethics Committee (Approval No. 2025KY154). Informed consent was waived due to the retrospective design of the study and the anonymization of patient data. A total of 219 breast cancer patients scheduled for TIVAP implantation via the IJV or AxV approach between September 2020 and February 2025 were assessed for eligibility. Six patients met exclusion criteria: axillary vascular anomalies (n = 1), IJV thrombosis history (n = 2), bilateral breast cancer (n = 2), and local infection (n = 1). Consequently, 213 patients were enrolled and allocated to either the IJV group (n = 109) or the AxV group (n = 104). All patients received the allocated port implantation procedure. Patients underwent a 6-month follow-up post-implantation. During follow-up, three patients in the IJV group and two patients in the AxV group were lost to follow-up. Therefore, the final analysis included 106 patients in the IJV group and 102 patients in the AxV group (**Figure 1**).

The inclusion criteria were as follows: age ≥ 18 years, feasible IJV or AxV access, histologically confirmed diagnosis of breast cancer, indication for chemotherapy according to the latest National Comprehensive Cancer Network (NCCN) guidelines, and ability to complete validated comfort, cosmetic outcome, and satisfaction questionnaires.

The exclusion criteria included the following: vascular anomalies in the neck/axillary regions, history of IJV/AxV thrombosis in the IJV or AxV, bilateral breast cancer, local infection or tumor invasion at the puncture site, and incomplete follow-up data. Data were collected via the electronic medical record system or telephone follow-ups to ensure the completeness and accuracy of case information. All case report forms were reviewed by a second investigator for completeness; electronic data entry was performed by two independent research assistants, with discrepancies resolved by recourse to the original sources; and range checks for data values were conducted prior to statistical analysis.

### 2.2 Variables

The exposure variable was venous access approach (ultrasound-guided IJV vs. AxV). The primary outcome variables were comfort levels evaluated at 1 day post-operation, as well as cosmetic outcomes and patient satisfaction assessed at 6 months post-implantation. Patients self-reported comfort using a standardized questionnaire. Cosmetic outcomes and overall satisfaction were similarly assessed using dedicated patient feedback questionnaires. PRO measures were collected at 1 day post-operation and during the 6-month follow-up by research assistants masked to group allocation. For the follow-up assessments (satisfaction and cosmetic outcome), assessors remained unaware of the allocated access route to minimize detection bias. Most interviews were conducted through structured telephone calls, while the remaining assessments took place during routine clinic visits, where matured scars unavoidably revealed group assignment. To enhance the efficacy of blinding, a standardized protocol was employed. Interviewers had no access to clinical records, and a neutral telephone script avoided any reference to surgical approaches. Additionally, patients were instructed prior to the call not to describe scar location, and a predefined unblinding protocol mandated that any accidental disclosure led to rescheduling with a different interviewer. Additionally, the following covariates were included: age, body mass index (BMI), tumor laterality, implantation side, previous catheter placement, and the use of antiplatelet or anticoagulant medications. Missing data were addressed using multiple imputation, with the missing data proportion below 10% to minimize the impact on results.

#### 2.3 Vein access selection criteria

The choice between IJV and AxV access was determined by the operating surgeon following a preoperative ultrasound assessment. Standardized criteria included the following: vein patency and diameter, anatomical accessibility (e.g., obesity, short neck, and prior surgery), requirement for contralateral access relative to the breast tumor side, and patient preference regarding cosmetic outcomes when vein anatomy was suitable for either approach. Both techniques were routinely available throughout the study period. Temporal analysis confirmed no significant trend in approach preference over time (Cochran–Armitage test, p = 0.312).

### 2.4 Port implantation procedure

To mitigate the potential impact of the learning curve associated with the AxV approach, all implantations in this study were performed by a dedicated two-surgeon team after they had surpassed the initial learning phase. Prior to the commencement of this cohort study, both surgeons had collectively performed more than 30 cases of ultrasound-guided AxV TIVAP implantations as part of their training and procedural standardization. This pre-study experience ensured that the technical proficiency and consistency of the AxV procedure were well-established before patient enrollment. Furthermore, all procedures in both groups were performed under continuous real-time ultrasound guidance, which is known to flatten the learning curve for venous access, with the patient in the supine position and the ipsilateral shoulder elevated. The target vein (IJV or AxV) was mapped using a high-resolution linear transducer (10–15 MHz) under sterile conditions. Ultrasound (US)-guided puncture was performed at a 30°–45° angle using a high-frequency linear transducer (12L, Siemens Acuson P500, Erlangen, Germany). The access site was always contralateral to the index breast lesion. A full surgical scrub and standard sterile draping were applied in every case, and all procedures were carried out under local anesthesia (1% lidocaine) with continuous real-time ultrasound guidance.

IJV approach: Under direct ultrasound visualization, the IJV was punctured with an 18-gauge needle. After free venous return, a 0.035-inch guidewire was advanced through the IJV and brachiocephalic vein into the superior vena cava (SVC). Tip position was verified using intraoperative digital subtraction angiography (DSA); malpositioned wires were repositioned under fluoroscopic control. A 3-cm transverse incision was then made 2 cm below the mid-clavicle to create a subcutaneous pocket. A tunneling device was used to draw the catheter from the pocket to the venotomy site. The catheter was trimmed to length and connected to the port, and the assembly was implanted in the pocket.

AxV approach: The patient was positioned supine with the arm abducted 90° angle. Imaging was conducted just distal to the costoclavicular space using a 10–15-MHz linear transducer. In the short-axis view, the vein lies deep to the pectoralis major and minor muscles; it was distinguished from the adjacent axillary artery by its compressibility and lack of pulsatility. The puncture was performed 1–2 cm lateral to the coracoid process and medial to the pectoralis minor tendon to avoid subsequent muscular compression of the catheter. Under real-time ultrasound guidance, an 18-gauge needle was advanced at a 30°–45° angle until free venous blood return was observed; a 0.035-inch guidewire was then advanced into the SVC under fluoroscopic guidance. A 2.5-cm lateral incision was made medial to the wire entry point, and the guidewire was externalized through the incision. Blunt dissection created a subcutaneous pocket of appropriate dimensions. The port was inserted directly into the pocket, and the catheter was trimmed to the appropriate length. Correct placement and free blood withdrawal were confirmed before wound closure with interrupted 4–0 absorbable sutures.

Identical titanium ports (4.8 Fr; PFM Medical, Cologne, Germany) were used in all patients. The target catheter tip position was the upper right atrium, with precise cavo-atrial junction localization verified using DSA in every case. Prophylactic antibiotics were not administered. Postoperatively, all patients received standardized care, including monthly flushing with heparinized saline.

Surveillance for catheter-related thrombosis was performed using a standardized ultrasound protocol. Patients with clinical symptoms (e.g., arm swelling and pain) underwent immediate duplex ultrasonography. Additionally, systematic screening with duplex ultrasonography was performed at 1 and 6 months post-implantation, or as clinically indicated. Thrombosis was defined as the presence of a non-compressible venous segment with or without a visible thrombus on B-mode and the absence of flow on Doppler interrogation.

### 2.5 Comfort, satisfaction, and cosmetic outcome assessments

The PRO measures used in this study, while disease-specific, have established validation and relevance in venous access research.

#### 2.5.1 Comfort assessment

Postoperative comfort was assessed at 1 day using a validated 6-point ordinal scale (Grades 0–5), which has demonstrated clinical validity and reliability in discriminating discomfort between venous access routes in prior randomized controlled trials and comparative studies (1, 12–14). The scale is defined as follows: Grade 0, without any discomfort; Grade 1, extremely mild discomfort; Grade 2, a little discomfort; Grade 3, some discomfort; Grade 4, rather uncomfortable; and Grade 5, extreme discomfort.

#### 2.5.2 Cosmetic outcome assessment

**Figure 2:**
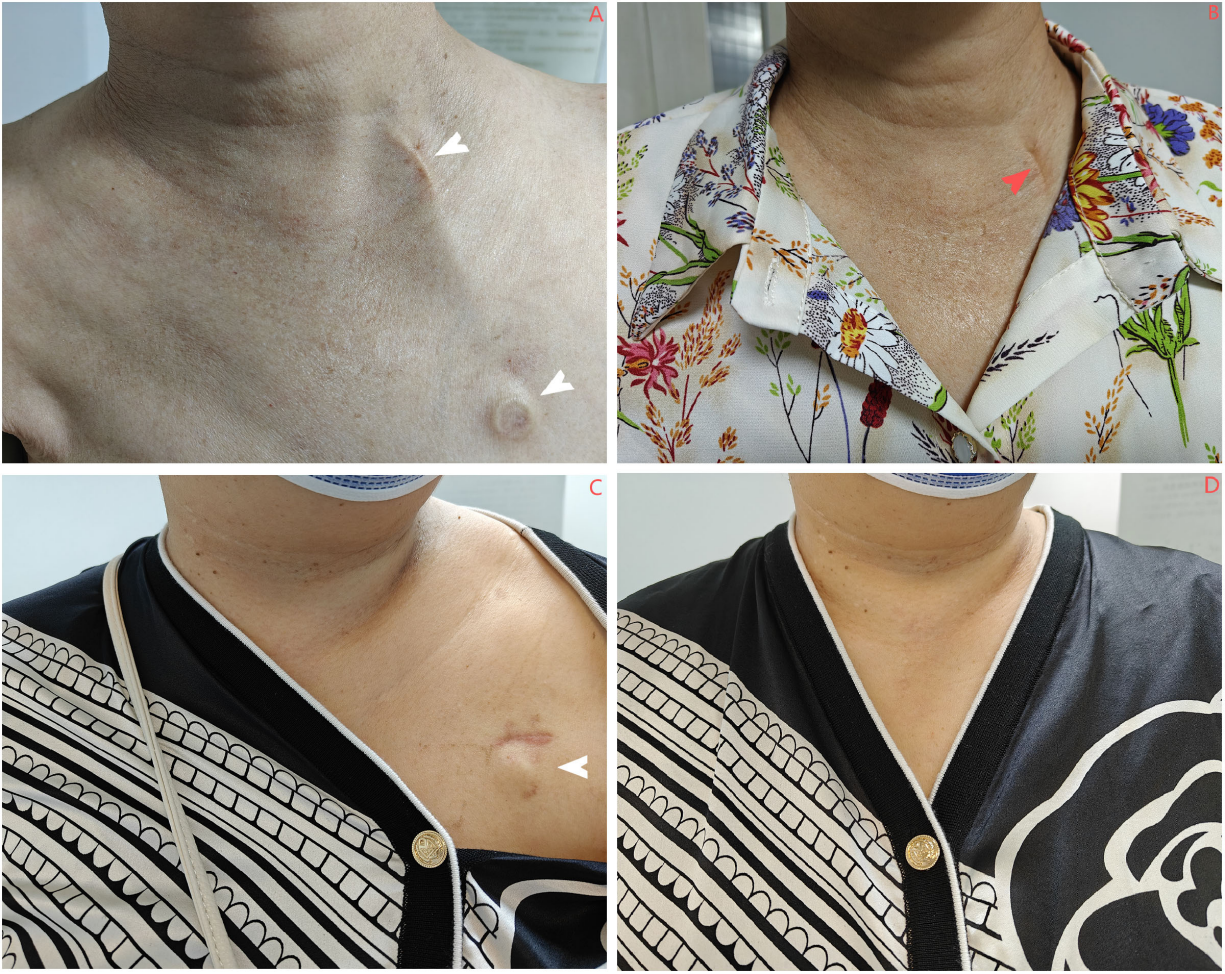
**(A, B)** Patients in the IJV group exhibit scars at the chest wall port site that can be effectively concealed by clothing (indicated by the white arrow). However, the catheter mark on the neck is quite prominent (denoted by the red arrow). **(C, D)** In contrast, patients in the AxV group show that the scars at the chest wall port site are also covered by clothing (indicated by the white arrow), and no surgical scars are visible. IJV, internal jugular vein; AxV, axillary vein.

Cosmetic results were evaluated at 6 months using a clinician-validated 4-grade system with established face and criterion validity in comparative TIVAP studies (12, 15, 16). This standardized tool focuses on changes in dressing habits, a key patient-centered cosmetic concern, and is defined as follows: Grade 1, comfortable and no need to change dressing habit; Grade 2, comfortable but needed to wear a polo shirt; Grade 3, comfortable but needed to button the top button of the polo shirt; and Grade 4, difficulty in dressing (**Figure 2**).

#### 2.5.3 Satisfaction assessment

Patient satisfaction was assessed at 6 months via a structured telephone interview using a standard 5-point Likert scale, a psychometrically validated and widely used instrument for measuring healthcare satisfaction (16). Patients rated their agreement with statements regarding overall satisfaction, willingness to choose the port again, and satisfaction with the cosmetic result (**Supplementary Material 1**). Responses were scored from 1 (“not at all satisfied”) to 5 (“very satisfied”); scores of 4 (“quite satisfied”) or 5 (“very satisfied”) were classified as “satisfied”.

### 2.6 Sample size calculation

Sample size estimation was based on a pilot study involving 30 patients (15 per group), not included in the final cohort, which demonstrated an absolute difference of 15% in the proportion of patients reporting Grade 0 comfort (no discomfort) at 1 day postoperatively. To detect this difference with a two-sided α = 0.05 and 80% power, accounting for an anticipated 10% dropout rate, a minimum of 100 patients per group was required. *Post-hoc* power analysis indicated 99% power to detect the observed 16.9% difference in Grade 0 comfort rates in our final cohort (IJV: 106, AxV: 102).

### 2.7 Statistical analysis

Continuous variables were presented as mean ± standard deviation (SD) and statistically compared using independent t-tests, following rigorous verification of data normality via the Shapiro–Wilk test and variance homogeneity assessment using Levene’s test; categorical variables were presented as frequencies and percentages [n (%)] and analyzed using chi-square or Fisher’s exact test, where applicable. Missing data for covariates and outcomes were handled using multiple imputation by chained equations (MICE) with 10 iterations. The only variables with missing values were BMI (1.9%), the 6-month cosmetic outcome score (2.4%), and the overall satisfaction score (1.4%). The proportion of missing data was <5% for all variables included in the imputation model, minimizing potential bias. Furthermore, to assess the robustness of the primary outcomes against potential confounding, multivariable logistic regression analyses were performed *post-hoc* for the endpoints of postoperative ideal comfort (Grades 0–1), favorable cosmetic outcome (Grades 1–2), and overall satisfaction. These models were adjusted for age, BMI, and implantation side. Complication rates were calculated as incidence density per 1,000 central line-days (CD), with the total observation period fixed at 6 months (180 days) for all patients. Group comparisons were performed using exact tests based on Poisson’s distribution. A significance level of p < 0.05 was considered statistically significant. Analyses were performed using SPSS version 27 (IBM Corp., Armonk, NY, USA).

## 3 Results

This retrospective study included 208 breast cancer patients who underwent port implantation via the IJV (n = 106) or AxV (n = 102). The groups were comparable in baseline characteristics: mean age (IJV: 54.7 ± 7.2 vs. AxV: 52.9 ± 6.6 years; p = 0.062), BMI (23.1 ± 1.5 vs. 22.7 ± 1.8 kg/m^2^; p = 0.083), implantation side (left: 48.1% vs. right: 51.0%; p = 0.339), and medical history (prior catheterization: 0.9% vs. 2.0%; p = 0.486; antiplatelet/anticoagulants: ≤3.9%; p > 0.479). The two groups were well-balanced with respect to all baseline characteristics (**Table 1**, all p > 0.05).

**Table 1:** Baseline characteristics of breast cancer patients undergoing TIVAP implantation via IJV versus AxV approach.

Characteristic	IJV group (n = 106)	AxV group (n = 102)	P-value
Age (years), mean ± SD	54.7 ± 7.2	52.9 ± 6.6	0.062
BMI (kg/m^2^), mean ± SD	23.1 ± 1.5	22.7 ± 1.8	0.083
Implantation side (%)			
Left	51 (48.1)	53 (51.0)	0.339
Right	55 (51.9)	49 (49.0)	0.339
Prior catheterization, n (%)	1 (0.9)	2 (2.0)	0.486
Antiplatelet use, n (%)	3 (2.8)	4 (3.9)	0.479
Anticoagulants, n (%)	3 (2.8)	2 (2.0)	0.518

Age and BMI were analyzed using independent t-tests. Data are n (%). Categorical variables were compared using the chi-square test or Fisher’s exact test.

TIVAP, totally implantable venous access port; IJV, internal jugular vein; AxV, axillary vein; BMI, body mass index.

Patients receiving AxV access demonstrated superior early postoperative comfort compared to those receiving the IJV approach (**Table 2**). The AxV cohort achieved significantly higher rates of complete comfort (Grade 0: 72.5% vs. 59.4% for IJV; p = 0.032), with an absolute difference of +13.1%. Notably, isolated minor discomfort (Grade 1) was more frequently reported with AxV access (17.7%) than IJV access (8.5%; p = 0.039). Critically, the composite rate of clinically ideal comfort (Grades 0–1) favored AxV access [90.2% (92/102) vs. 67.9% (72/106)], establishing a 22.3% absolute advantage in symptom-free recovery.

**Table 2:** Postoperative comfort scores at 1 day by implantation approach.

Patient comfort grade	IJV group (n = 106)	AxV group (n = 102)	P-value
Grade 0	63 (59.4)	74 (72.5)	0.032
Grade 1	9 (8.5)	18 (17.7)	0.039
Grade 2	24 (22.6)	10 (9.8)	0.01
Grade 3	10 (9.5)	0	<0.001
Grade 4	0	0	–
Grade 5	0	0	–

Data are n (%). Group comparisons were performed using the chi-square test or Fisher’s exact test.

IJV, internal jugular vein; AxV, axillary vein.

At the 6-month follow-up, AxV access showed significantly better cosmetic outcomes compared to IJV access (**Table 3**). Favorable cosmetic outcomes (Grades 1–2) were significantly more prevalent in the AxV group (93.1% vs. 67.9%; p < 0.001).

**Table 3:** Cosmetic outcomes at 6-month follow-up by implantation approach.

Cosmetic outcome	IJV group (n = 106)	AxV group (n = 102)	P-value
Grade 1	43 (40.5)	55 (53.9)	0.037
Grade 2	29 (27.4)	40 (39.2)	0.048
Grade 3	23 (21.7)	5 (4.9)	<0.001
Grade 4	11 (10.4)	2 (2.0)	<0.001
Favorable outcome (Grades 1–2)	72 (67.9)	95 (93.1)	<0.001

Data are n (%). Group comparisons were performed using the chi-square test or Fisher’s exact test.

IJV, internal jugular vein; AxV, axillary vein.

At the 6-month follow-up, patient satisfaction was significantly higher for AxV access compared to IJV access (**Table 4**). Overall satisfaction, defined as reporting “very” or “quite” satisfied, was observed in 94.1% of AxV patients, compared to 85.8% in the IJV group (p = 0.039; absolute difference +8.3%). Furthermore, 83.3% of AxV patients indicated they would opt for the port again versus 72.6% in the IJV group (p = 0.045; absolute difference +10.7%). These results highlight the superior long-term acceptance and satisfaction associated with the AxV approach for port implantation in breast cancer patients.

**Table 4:** Patient satisfaction at 6-month follow-up by implantation approach.

Satisfaction measure	IJV group (n = 106)	AxV group (n = 102)	P-value
Satisfaction, n (%)			
Not at all satisfied	4 (3.8)	0 (0)	0.055
A little satisfied	5 (4.7)	2 (2.0)	0.243
Somewhat satisfied	6 (5.7)	4 (3.9)	0.576
Quite satisfied	75 (70.7)	29 (28.4)	<0.001
Very satisfied	16 (15.1)	67 (65.7)	<0.001
“Very”/”quite” satisfied	91 (85.8)	96 (94.1)	0.039
Willingness to choose port again, n (%)			
“Very”/”quite” likely	77 (72.6)	85 (83.3)	0.045

Satisfaction was defined as “very”/”quite”. Data are n (%). Group comparisons were performed using the chi-square test or Fisher’s exact test.

IJV, internal jugular vein; AxV, axillary vein.

Multivariable logistic regression analyses, adjusting for age, BMI, and implantation side, confirmed the independent benefits of the AxV approach across all primary outcomes (**Table 5**). Compared to the IJV approach, the AxV approach remained independently and significantly associated with higher odds of ideal postoperative comfort [adjusted odds ratio (aOR) = 4.48, 95% CI: 2.04–9.85, p < 0.001], favorable cosmetic outcome (aOR = 6.22, 95% CI: 2.59–15.00, p < 0.001), and overall satisfaction (aOR = 3.07, 95% CI: 1.09–8.60, p = 0.033). None of the adjusted covariates demonstrated significant associations with the outcomes. All regression models demonstrated adequate fit (Hosmer–Lemeshow test p > 0.05).

**Table 5:** Multivariable logistic regression analysis for primary outcomes.

Outcome measure	Variable	Adjusted odds ratio (aOR)	95% confidence interval	P-value
Ideal comfort (Grades 0–1)	AxV approach	4.48	2.04–9.85	**<0.001**
	Age	1.03	0.97–1.08	0.372
	BMI	0.82	0.66–1.03	0.084
	Implantation side (left)	0.77	0.38–1.57	0.475
Favorable cosmetic outcome (Grades 1–2)	AxV approach	6.22	2.59–15.00	**<0.001**
	Age	0.98	0.93–1.04	0.532
	BMI	0.99	0.79–1.25	0.946
	Implantation side (left)	1.15	0.56–2.38	0.704
Overall satisfaction (“quite/very”)	AxV approach	3.07	1.09–8.60	**0.033**
	Age	1.01	0.94–1.08	0.812
	BMI	1.31	0.97–1.78	0.076
	Implantation side (left)	0.97	0.38–2.44	0.945

The reference group for the venous access approach is the IJV group. aOR > 1 indicates a higher odds of the outcome for the AxV group. Bolded values indicate statistical significance (p < 0.05).

AxV, axillary vein; BMI, body mass index.

The overall incidence of procedure-related complications within 6 months was low and comparable between the IJV (4.8%, 0.269/1,000 CD) and AxV (4.9%, 0.279/1,000 CD) groups (p = 0.957). No statistically significant differences were observed for any specific complication type (**Table 6**, all p > 0.05). In the IJV group, one patient developed a catheter-related bloodstream infection (CRBSI) at 1 month. The infection was not controlled with antibiotics, ultimately leading to unplanned removal of the TIVAP.

**Table 6:** Incidence density of procedure-related complications within 6 months by implantation approach.

Complication type	IJV group (n = 106, 18,570 CD)		AxV group (n = 102, 17,950 CD)		P-value
	Events (n, %)	Rate (per 1,000 CD)	Events (n, %)	Rate (per 1,000 CD)	
Infection of the dermal pocket	0 (0)	0	0 (0)	0	–
Catheter-related bloodstream infection (CRBSI)	1 (0.9)	0.054	0 (0)	0	0.326
Asymptomatic catheter-related thrombosis	3 (2.8)	0.161	4 (3.9)	0.223	0.672
Symptomatic venous thrombosis	0 (0)	0	1 (1.0)	0.056	0.309
Catheter migration	0 (0)	0	0 (0)	0	–
Unplanned removal	1 (0.9)	0.054	0 (0)	0	0.326
Total complications	5 (4.8)	0.269	5 (4.9)	0.279	0.957

Data are presented as number of events (percentage) and incidence density (per 1,000 central venous days). Group comparisons were performed using exact tests based on Poisson’s distribution for incidence rates. The total observation period was fixed at 6 months (180 days) for all patients, with follow-up censored at the time of complication occurrence for affected patients.

CD, central venous days; IJV, internal jugular vein; AxV, axillary vein.

## 4 Discussion

This single-center retrospective cohort study (n = 208) provides compelling evidence demonstrating that the ultrasound-guided AxV approach for TIVAP implantation in breast cancer patients yields superior PROs compared to the IJV approach. Specifically, patients undergoing AxV access experienced significantly higher rates of early postoperative comfort (Grade 0: 72.5% vs. 59.4%; p = 0.032; Grade 1: 17.7% vs. 8.5%; p = 0.039), more favorable long-term aesthetic outcomes (Grades 1–2: 93.1% vs. 67.9%; p < 0.001), and greater overall satisfaction at 6 months (94.1% vs. 85.8%; p = 0.039). Of patients in the AxV group, 83.3% indicated their willingness to choose the port again versus 72.6% in the IJV group (p = 0.045). Crucially, these benefits were observed without evidence of a significant increase in the risk of periprocedural or early complications within the 6-month follow-up period, as both approaches demonstrated comparable overall complication rates (4.8%, 0.269/1,000 CD vs. 4.9%, 0.279/1,000 CD; p = 0.957).

Our results showing significantly better comfort, cosmetic outcomes, and satisfaction with the AxV approach are consistent with those of previous studies. Consistent with our findings, prior randomized controlled studies (1) have reported reduced early postoperative pain and discomfort with AxV/SCV approaches compared to IJV access. Another study also showed that the pain and discomfort associated with AxV access for TIVAP were worse than those associated with IJV access (17). Previous studies on posterior IJV modification reported favorable aesthetic outcomes: 23.9% of patients required no clothing adjustments, while 76.1% needed only collared garments to conceal the catheter course (12). Although the posterior approach positions the subcutaneous segment along the posterior cervical triangle—where it remains concealed by clothing and minimizes kinking through gentle curvature—the novel single-incision AxV technique eliminates tunneling entirely. This technique yields a discreet infraclavicular scar without restricting clothing options. The superior cosmetic outcome and unrestricted clothing choices afforded by the AxV approach are particularly valued by women aiming to maintain their social and professional activities and body image after breast cancer diagnosis. The AxV cohort demonstrated significantly higher satisfaction (94.1% vs. 85.8%; p = 0.039), with 83.3% indicating preference for reimplantation via the same approach, confirming superior patient acceptance. The AxV approach is associated with significantly better postoperative outcomes compared to the IJV approach, including higher odds of ideal comfort (aOR = 4.48, p = 0.0002), improved cosmetic results (aOR = 6.22, p < 0.0001), and enhanced overall satisfaction (aOR = 3.07, p = 0.0331). None of the adjusted covariates showed significant associations with these outcomes, highlighting the advantages of the AxV approach.

The enhanced patient-reported comfort with AxV access is mechanistically attributable to its tunnel-less design requiring only a single infraclavicular incision. Conventional IJV access requires the creation of a subcutaneous tunnel connecting the venipuncture site in the neck to the port pocket in the infraclavicular region. The skin and subcutaneous tissues of the neck, particularly the platysma muscle, are dissected during IJV tunneling, traversing the transverse cervical nerve and supraclavicular nerves, which densely innervate the platysma and cervical skin. These nerves are primarily responsible for superficial sensations. Procedures, especially the creation of subcutaneous tunnels, inevitably irritate or damage these fine nerve fibers, leading to postoperative pain, paresthesia, and even a feeling of traction. Additionally, the inflammatory response of the surrounding tissues may still indirectly stimulate these nerves (18). The tunnel may lead to a persistent foreign-body sensation, which is exacerbated by friction and movement of the overlying skin and muscles (8). By accessing the axillary vein distal to the costoclavicular space, the AxV approach circumvents clavicular compression—a key risk factor for pinch-off syndrome—while eliminating tunneling (6, 17). The single incision, strategically placed within the infraclavicular crease, heals inconspicuously. Furthermore, eliminating the subcutaneous neck tunnel reduces visible catheter ridging and palpability under the skin, which was a significant contributor to unfavorable cosmetic outcomes and clothing restrictions in the IJV group. This enhanced cosmesis and freedom in clothing choice (particularly avoiding high-necked garments to conceal neck scars/tunnels) are of paramount importance for body image and social reintegration in women undergoing breast cancer treatment.

The comparable 6-month complication rates (IJV: 4.8% vs. AxV: 4.9%; p = 0.602) align with contemporary benchmarks (2, 19, 20). A prior retrospective analysis (21) comparing IJV and AxV approaches likewise reported no significant inter-group differences in long-term morbidity—thrombosis (2.5% vs. 0%, p = 0.444) and infection (2.5% vs. 2.0%, p = 1.000) were infrequent in both cohorts. Conversely, a prospective randomized trial (8) observed a numerically higher incidence of catheter-related thrombosis in the AxV group (risk ratio 2.60; 95% CI: 0.53–12.61), although the difference did not reach statistical significance. Our findings showed no significant difference between the IJV and AxV approaches. This comparably low rate in both groups may be attributed to several factors: the use of small-bore (4.8 Fr) catheter systems, which are associated with reduced thrombogenicity (22); meticulous ultrasound-guided puncture minimizing endothelial trauma; and precise catheter tip positioning confirmed by intraoperative DSA at the cavoatrial junction (23), optimizing flow dynamics. As shown in previous studies (6), a CRBSI is a significant cause of premature removal of TIVAP. In our study, one patient (0.9%) in the IJV group developed a CRBSI at 1 month postoperatively, leading to the unplanned removal of the TIVAP. This is consistent with the incidence reported in the literature (17). Immediate and early complications were not the focus of our study, but according to previous reports (17), compared with the IJV approach, the single-incision AxV puncture technique significantly reduced the operative time, the incidence of localized ecchymosis, and immediate adverse events, while early complications were comparable.

Several limitations merit acknowledgment. First, the single-center retrospective design inherently limits causal inference due to potential selection bias arising from non-random treatment allocation; prospective, multicenter randomized controlled trials are warranted to validate these associations and eliminate selection bias. Second, our findings on safety are confined to the early and mid-term post-implantation periods; the follow-up period was limited to 6 months. This precludes the assessment of important late complications such as delayed catheter-related thrombosis, catheter fracture/migration, or long-term port failure rates, which may differ between approaches. Third, the study cohort comprised exclusively female breast cancer patients; extrapolation to other oncologic or non-oncologic populations necessitates further validation. Fourth, although the PRO measures used in this study were derived from previously published and applied tools in the field of venous access, they are not internationally standardized questionnaires. The future incorporation of fully validated and generic PRO instruments could further strengthen the comparability of such outcomes across different studies. Due to the retrospective design, we were unable to systematically capture certain clinically relevant variables, such as prior breast surgery, chest wall radiotherapy, or pre-existing neck/shoulder pain. These factors were rarely documented (≤3%) and could not be meaningfully analyzed. We recognize this as a limitation and plan to prospectively collect these variables in future studies.

## 5 Conclusion

For breast cancer patients receiving TIVAPs, ultrasound-guided AxV access is associated with significant advantages, including enhanced early postoperative comfort, superior long-term cosmetic results, and elevated patient satisfaction, all achieved without compromising procedural safety or increasing early complication risks within the first 6 months post-intervention.

## Data Availability

The datasets used and/or analysed during the current study are available from the corresponding author upon reasonable request. Requests to access these datasets should be directed to zhantenghui@fjsfy.com.

## References

[1] Chen YB Bao HS Hu TT He Z Wen B Liu FT . Comparison of comfort and complications of Implantable Venous Access Port (IVAP) with ultrasound guided Internal Jugular Vein (IJV) and Axillary Vein/Subclavian Vein (AxV/SCV) puncture in breast cancer patients: a randomized controlled study. BMC Cancer. (2022) 22:248. doi: 10.1186/s12885-022-09228-6, PMID: 35248019 PMC8898472

[2] Luo H Pan Y Zhu S Huang L Dong M Zhang Q . Tunnelless totally implantable venous access device implantation via ultrasound-guided axillary vein puncture: A retrospective analysis. J Int Med Res. (2025) 53:3000605251357876. doi: 10.1177/03000605251357876, PMID: 40686261 PMC12280531

[3] Caiazzo M Golino L Addeo R Fardello F Russo G Imperatore F . A delayed complication of a port-a-cath insertion via subclavian venous access: Case report of a “pinch-off syndrome. Int J Surg Case Rep. (2022) 94:107039. doi: 10.1016/j.ijscr.2022.107039, PMID: 35461178 PMC9046788

[4] Tabatabaie O Kasumova GG Eskander MF Critchlow JF Tawa NE Tseng JF . Totally implantable venous access devices. Am J Clin Oncol. (2017) 40(1):94–105. doi: 10.1097/COC.0000000000000361, PMID: 28106685

[5] Velioğlu Y . Complications and management strategies of totally implantable venous access port insertion through percutaneous subclavian vein. Turk J Thorac Cardiovasc Surg. (2019) 27(4):499–507. doi: 10.5606/tgkdc.dergisi.2019.17972, PMID: 32082916 PMC7018162

[6] Seo TS Song MG Kim JS Choi CW Seo JH Oh SC . Long-term clinical outcomes of the single-incision technique for implantation of implantable venous access ports via the axillary vein. J Vasc Access. (2017) 18:345–51. doi: 10.5301/jva.5000751, PMID: 28665466

[7] Shinde P Jasapara A Bansode K Bunage R Mulay A Shetty V . A comparative study of safety and efficacy of ultrasound-guided infra-clavicular axillary vein cannulation versus ultrasound-guided internal jugular vein cannulation in adult cardiac surgical patients. Ann Card Anaesth. (2019) 22(2):177–86. doi: 10.4103/aca.ACA_24_18, PMID: 30971600 PMC6489407

[8] Pignataro BS Nishinari K Krutman M Ymagawa Fonseca IY Cavalcante RN Centofanti G . Axillary versus jugular vein for totally implanted port-A-cath, randomized, controlled trial. Ann Vasc Surg. (2025) 117:56–63. doi: 10.1016/j.avsg.2025.03.035, PMID: 40246275

[9] Han L Zhang J Deng X Kong X Yang C Peng L . Totally implantable venous access ports: A prospective randomized study comparing subclavian and internal jugular vein punctures in children. J Pediatr Surg. (2021) 56:317–23. doi: 10.1016/j.jpedsurg.2020.04.021, PMID: 32467037

[10] Li Q Peng X Leng X Xiao S Min M Wan S . Comparing internal jugular vein and subclavian vein for central venous insertion of implantable ports in cancer chemotherapy: a meta-analysis of RCTs. Front Oncol. (2025) 15:1566757. doi: 10.3389/fonc.2025.1566757, PMID: 40492120 PMC12146329

[11] Zhu L Li K He Q Liu L . Psychological experiences and needs of tumor patients with implanted intravenous infusion ports: a qualitative study. Front Oncol. (2024) 14:1392416. doi: 10.3389/fonc.2024.1392416, PMID: 38817894 PMC11137243

[12] Wang M Tang L Xu R Qin S Zhang S . Clinical application of ultrasound-guided totally implantable venous access ports implantation via the posterior approach of the internal jugular vein. J Chin Med Assoc. (2024) 87(1):126–30. doi: 10.1097/JCMA.0000000000001030, PMID: 38016115 PMC12718871

[13] Mauri T Galazzi A Binda F Masciopinto L Corcione N Carlesso E . Impact of flow and temperature on patient comfort during respiratory support by high-flow nasal cannula. Crit Care. (2018) 22:120. doi: 10.1186/s13054-018-2039-4, PMID: 29743098 PMC5941611

[14] Babu KG Suresh Babu MC Lokanatha D Bhat GR . Outcomes, cost comparison, and patient satisfaction during long-term central venous access in cancer patients: Experience from a Tertiary Care Cancer Institute in South India. Indian J Med Paediatr Oncol. (2016) 37(4):232–8. doi: 10.4103/0971-5851.195732, PMID: 28144088 PMC5234158

[15] Cheng HW Ting CK Chu YC Chang WK Chan KH Chen PT . Application of an ultrasound-guided low-approach insertion technique in three types of totally implantable access port. J Chin Med Assoc. (2014) 77(5):246–52. doi: 10.1016/j.jcma.2014.02.005, PMID: 24726676

[16] Nagel SN Teichgräber UK Kausche S Lehmann A . Satisfaction and quality of life: a survey-based assessment in patients with a totally implantable venous port system. Eur J Cancer Care (Engl). (2011) 21(2):197–204. doi: 10.1111/j.1365-2354.2011.01275.x, PMID: 21851433

[17] Mu C Zhu Z Miao D Wu Q Chen L Jin Y . Clinical efficacy and safety of a new single-incision axillary vein puncture technique for totally implantable venous access ports. Sci Rep. (2018) 15:7281. doi: 10.1038/s41598-025-91827-x, PMID: 40025108 PMC11873167

[18] Jarvis MS Sundara Rajan R Roberts AM . The cervical plexus. J Natl Compr Canc Netw. (2023) 23(2):46–51. doi: 10.1016/j.bjae.2022.11.008, PMID: 36686890 PMC9845551

[19] Kim DH Ryu DY Jung HJ Lee SS . Evaluation of complications of totally implantable central venous port system insertion. Exp Ther Med. (2019) 17:2013–18. doi: 10.3892/etm.2019.7185, PMID: 30867691 PMC6395957

[20] Abou-Mrad A Marano L Oviedo RJ . A monocentric analysis of implantable ports in cancer treatment: five-year efficacy and safety evaluation. Cancers. (2024) 16(16):2802. doi: 10.3390/cancers16162802, PMID: 39199575 PMC11352375

[21] Tian K Jiang J Liu D Zhou H Zhou L Deng K . The advantages and safety of ultrasound-guided infusion port implantation via axillary vein. Ann Vasc Surg. (2025) 118:161–8. doi: 10.1016/j.avsg.2025.05.004, PMID: 40398810

[22] Streiff MB Holmstrom B Ashrani A Bockenstedt PL Chesney C Eby C . Cancer-associated venous thromboembolic disease, version 1.2015. Ann Oncol. (2015) 13:1079–95. doi: 10.6004/jnccn.2015.0133, PMID: 26358792

[23] Sousa B Furlanetto J Hutka M Gouveia P Wuerstlein R Mariz JM . Central venous access in oncology: ESMO Clinical Practice Guidelines. Ann Oncol. (2015) 26:v152–68. doi: 10.1093/annonc/mdv296, PMID: 26314776

