# Supplementary material for: Ultrasound-guided axillary vein versus internal jugular vein access for totally implantable venous access ports in breast cancer: a retrospective comparison of patient-reported outcomes
Source: Front Oncol. 2025 Nov 6;15:1684119. doi: 10.3389/fonc.2025.1684119 (PMC12630992; doi:10.3389/fonc.2025.1684119)
Supplement: Supplementary file 1 [file Table1.docx]

**Patient-reported **satisfaction questionnaire****

|  | 1 = 'not at all satisfied' | 2 = 'a little satisfied' | 3 = 'somewhat satisfied' | 4 = 'quite satisfied' | 5 = 'very satisfied' |
| --- | --- | --- | --- | --- | --- |
| Overall, how satisfied are you with the port system? |  |  |  |  |  |
| Satisfaction with the cosmetic result |  |  |  |  |  |
| Willingness to choose the port again |  |  |  |  |  |
